# Applied Machine Learning in Spiral Breast-CT: Can We Train a Deep Convolutional Neural Network for Automatic, Standardized and Observer Independent Classification of Breast Density?

**DOI:** 10.3390/diagnostics12010181

**Published:** 2022-01-13

**Authors:** Anna Landsmann, Jann Wieler, Patryk Hejduk, Alexander Ciritsis, Karol Borkowski, Cristina Rossi, Andreas Boss

**Affiliations:** Institute for Diagnostic and Interventional Radiology, University Hospital Zurich, Rämistrasse 100, 8091 Zürich, Switzerland; jann.wieler@usz.ch (J.W.); Patryk.Hejduk@usz.ch (P.H.); alexander.ciritsis@usz.ch (A.C.); karol.borkowski@usz.ch (K.B.); cristina.rossi@usz.ch (C.R.); andreas.boss@usz.ch (A.B.)

**Keywords:** spiral breast-CT, photon counting breast-CT, machine learning, artificial intelligence

## Abstract

The aim of this study was to investigate the potential of a machine learning algorithm to accurately classify parenchymal density in spiral breast-CT (BCT), using a deep convolutional neural network (dCNN). In this retrospectively designed study, 634 examinations of 317 patients were included. After image selection and preparation, 5589 images from 634 different BCT examinations were sorted by a four-level density scale, ranging from A to D, using ACR BI-RADS-like criteria. Subsequently four different dCNN models (differences in optimizer and spatial resolution) were trained (70% of data), validated (20%) and tested on a “real-world” dataset (10%). Moreover, dCNN accuracy was compared to a human readout. The overall performance of the model with lowest resolution of input data was highest, reaching an accuracy on the “real-world” dataset of 85.8%. The intra-class correlation of the dCNN and the two readers was almost perfect (0.92) and kappa values between both readers and the dCNN were substantial (0.71–0.76). Moreover, the diagnostic performance between the readers and the dCNN showed very good correspondence with an AUC of 0.89. Artificial Intelligence in the form of a dCNN can be used for standardized, observer-independent and reliable classification of parenchymal density in a BCT examination.

## 1. Introduction

Breast cancer (BC) constitutes the second most common cause of cancer death in women. With an incidence of 12.3% (1 in 8 women) in the female population, it is the most frequently diagnosed cancer among women and surpasses lung cancer as the most commonly diagnosed cancer worldwide [1]. Yet, while not established everywhere, mammography screening is known to reduce mortality in breast cancer and it has been estimated that the combination of mammography screening with adjuvant therapy results in a relative reduction in mortality in breast cancer patients of 37% [2]. Risk factors for breast cancer are diverse. Besides genetic predisposition, hormonal influences such as estrogen replacement therapy and oral contraception are discussed as potential risk factors for breast cancer [3]. Moreover, epidemiological studies have shown that the risk for breast cancer in women with a higher density of breast tissue may increase two to six times [4]. However, in spite of the clinical importance of the density of breast tissue, it is difficult to determine in a clinical examination [5].

Mammographic density (MD) is defined as the relative amount of glandular tissue based on the mammographic appearance of fibroglandular tissue on the mammogram. Breast density (BD) not only underlies the influence of age and menopausal transition, but also lifestyle factors such as body mass index, alcohol intake or breastfeeding are reflected in MD [3,6]. MD is usually classified according to the ACR BI-RADS atlas, last updated in 2013, classifying MD in four categories from A to D, with category A describing almost completely fatty tissue, B describing scattered fibroglandular tissue, C describing heterogenous dense tissue and D describing extremely dense tissue [7].

Besides its relevance in the assessment of the individual risk of developing breast cancer, MD represents an important parameter in the planning of systemic mammography screening programs. The sensitivity of screening mammograms strongly depends on MD: while for low density breasts, a sensitivity of 87% is reported, the sensitivity decreases to only 63% in dense breast tissue. Moreover, the assessment of breast density is indicative for the need of an additional ultrasound examination. Studies have shown that an additional ultrasound in women with MD “C” or “D” improves the cancer detection rate with additional 2–4 cancers per 1000 patients [3,8,9], which is therefore recommended in many guidelines [10].

One reason for the restricted sensitivity in dense breast tissue is overlay in a planar 2D projection image. Recently spiral breast-CT (BCT), using photon-counting detector technology, a truly 3D breast imaging modality, has been introduced as an alternative to mammography or breast-MRI in the use for mammography screening. BCT prevents the overlay of parenchyma, as observed in conventional mammograms, by the ability of multislice imaging and multiplanar reconstruction, while offering a high isotropic spatial resolution [11,12]. This also allows density measurements of detected lesions and contrast enhanced BCT using iv-injected iodinated contrast media, which might be an alternative for patients with contraindications to MR mammography [13].

Unlike common mammography and tomosynthesis, spiral breast-CT is a non-compression technique ensuring more comfort during image acquisition and therefore, a higher compliance in breast cancer screening by women [14]. Due to different X-ray absorption properties, glandular breast tissue, consisting of epithelial and stroma cells, appears hyperdense on a spiral breast-CT compared to fatty tissue, which appears hypodense. In this way, BCT provides an objective assessment of the relative amount of glandular tissue in the breast, based on a high-resolved, three-dimensional acquisition technique. Regarding lesion detection, the sensitivity of BCT in dense tissue was reported to be higher compared to conventional mammography at a comparable radiation dose [15,16]. Cone-beam CT, instead, is reported to exhibit a significantly higher radiation dose compared to mammography [16].

Existing studies on BCT regarding breast density use the ACR BI-RADS atlas to describe MD in BCT [17]. Yet, the BI-RADS atlas has been developed for conventional projection mammography and does not consider the three-dimensional nature of BCT imaging datasets. A new dedicated BCT breast density classification atlas has recently been proposed by Wieler et al. [18], implementing fatty septae into the breast density assessment, which are unique in BCT and allow for the improved distinction of lesions within parenchyma. In our study, breast density was assessed by human reading, using the provided atlas by Wieler et al. and we subsequently trained a deep convolutional neural network (dCNN) for automatic breast CT density (BCTD) classification. Recent projects have already successfully applied machine learning algorithms to analyse medical images [19]. For example, a dCNN has been used to successfully classify mammographic images regarding MD according to the ACR BI-RADS classification system [20]. In another study the same dCNN was used to detect and classify breast lesions in automated breast ultrasound with high accuracy [21]. Saffari et al. trained another deep learning algorithm using synthetic data generated by a conditional Generative Adversarial Network (cGAN). In their work, they were able to segmentate, fully automate and classify breast density in conventional mammograms with an accuracy of 98% [22]. Due to the high volume of a BCT examination and the resulting extended evaluation time, there is a need of automated breast density assessment. In this current study, we evaluated whether a deep convolutional neural network (dCNN) trained with 5589 images of 634 BCT examinations allows for accurate, objective and standardized BCTD classification. The aim of this study was to obtain an automated and observer-independent classification system for BCTD based on an ACR BI-RADS-like system.

## 2. Materials and Methods

### 2.1. Patient Selection

A retrospective analysis of patient data in the local PACS archive of our institution was performed and approved by the local ethics committee. All patients signed informed consent for the scientific evaluation of the imaging and clinical data. Imaging data from 520 BCT examinations between January and August 2021 were included in the study. Patients with prior surgery or radiation therapy of the breast (*n* = 198), contrast enhanced BCT (*n* = 2) exams using iv-injection of iodinated contrast-media, or unilateral examinations (*n* = 3) were excluded. Additional demographic data is not routinely documented in radiologic reports or in the PACS of our institution and was therefore not further analysed.

### 2.2. BCT Examinations

All examinations were performed on a dedicated spiral breast CT imager equipped with a CdTe photon-counting detector with an area of 280 × 50 mm^2^ (nu-view, AB-CT—Advanced Breast-CT GmbH, Erlangen, Germany). The maximum diameter of the Field-of-View (FOV) is 190 mm, and the scan length can be adjusted to the values 80, 120 and 160 mm, depending on the size of the breast, resulting in a 311, 450 or 588 slices BCT exam. The X-ray tube exhibits a 0.3 mm focal spot size, and a 3 mm AI filtration is applied. A fixed X-ray tube voltage of 60 kV is used, whereas the tube current may be adjusted between 5 mA and 125 mA. In all patients, a tube current of 32 mA was applied. No intra-venous injection of contrast media was applied in the retrospective study cohort. The examination scans were acquired in a spiral mode with the high-resolution scan protocol, with scan times of 7, 9.5 and 12 s for the scan length of 80, 120 and 160 mm, respectively. Image reconstruction was carried out in a standard mode with a soft kernel at 300 µm^3^ voxel size with 2 × 2-pixel binning, using a Feldkamp-type filtered back projection (FBP) algorithm.

### 2.3. Breast Density

**Figure 1:**
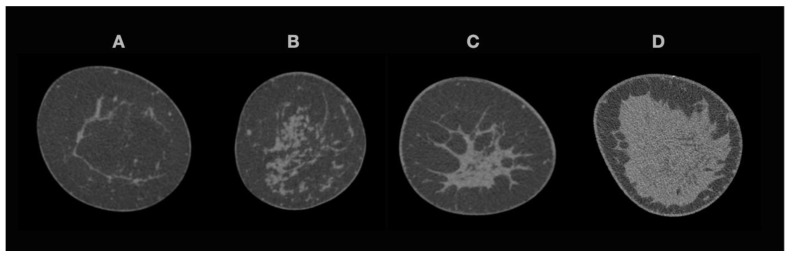
Spiral breast-CT density classification with one example for each category. (**A**). Partial or complete involution with every lesion visible; (**B**). Scattered glandular tissue with lesions larger than 10 mm conclusively visible; (**C**). Heterogenous dense glandular tissue with lesions of 10 mm potentially not visible; (**D**). Very dense tissue with restricted visibility of lesions. Raw data images with 0.3 mm slice thickness, coronal plane.

Human reading was performed by a radiology resident with one year of experience in mammographic imaging, regarding raw data images in the coronal plane. BCT examinations were categorized in four groups, with different breast densities based on lesion detectability, according to Wieler et al. [18], as depicted in Figure 1.

For the machine learning algorithm, only representative slices of the breast tissue were extracted from the BCT dataset to avoid confusion of the dCNN, reading empty (black) images, bony tissue, or pectoralis muscle tissue. Depending on the breast volume, the number of images from one patient examination included for the machine learning algorithm ranged between 40 and 200 images of each side.

### 2.4. Data Preparation

Data retrieval and annotation resulted in 55,896 images belonging to the four different classes of breast density. For further analysis, every tenth image was used, whereas 9 out of 10 images were omitted to reduce intra-individual dependence of data. The remaining 5589 images used for dCNN training or validation were resized from their initial dimensions either to a matrix size of 256 × 256 pixels or 512 × 512 pixels, depending on the subsequently applied dCNN architecture.

Data augmentation was performed with the ImageDataGenerator of Keras, expanding mammograms with a zoom factor of 0.1, a rotation of 45°, a horizontal shift factor of 0.1, and a vertical shift factor of 0.1. Brightness was kept constant in the data augmentation.

The resulting dataset was randomly shuffled and subsequently split into training, validation, and test data; 70% of the images were used for the training of the dCNN and 20% were used for the validation of the resulting model. In order to evaluate the performance of the dCNN, a “real world” dataset including 10% of the data not previously used, either for training or for validation, and spared from data augmentation, was created.

Additionally, to evaluate the unbiased performance of the dCNN a subset of the test dataset was generated, taking 60 images from the original test dataset, 15 images for each density category. The dCNN’s mammography density classification for the subset was compared to human reading performed by two experienced radiologists in breast-CT assessment.

### 2.5. dCNN Architecture and Training

All computations were performed on a desktop computer equipped with an Intel i7-9700 CPU with 16 GB RAM and NVIDIA RTX 1080 SUPER graphics processing unit with 8 GB graphics RAM. The desktop PC ran under Ubuntu 20.04 with Tensorflow 2.5.0. and Keras 2.4.3. All programming were performed in the computer language, Python (Version 3.8.5; Python Software Foundation, Wilmington, DE, USA).

Four dCNN models were generated classifying the previously described four levels of breast density, according to the provided classification system by Wieler et al., with differences in the spatial resolution, the optimizer and previous cropping of images: model 1 (input shape 512 × 512, optimizer Adam, no cropping), model 2 (input shape 256 × 256, optimizer Adam, no cropping), model 3 (input shape 512 × 512, optimizer SGD, no cropping), and model 4 (input shape 512 × 512, optimizer Adam, central cropping of image). Each of the models was a multilabel classifier distinguishing all BI-RADS classes together. Table 1 provides an overview of the four models and their characteristics.

The dCNN architecture consisted of thirteen convolutional layers followed by max-pooling and two dense layers with a final fully connected softmax layer. The convolutional layers were zero-padded and Nesteroy momentum and dropout of 0.5 were used to improve the performance of the model and to prevent overfitting. Batch size was set to 8 and the number of epochs for training was 160 in all four models created. The learning rate was set to 1 × 10^−5^ and the loss function was “cross entropy”. The architecture of our dCNN model is added as supplementary material.

All four models were trained at the same time. After complete training and validation of the model, density classification was assigned to each image of the “real world” test dataset, based on the highest probability assigned to the four categories A to D (keras predict_proba function).

### 2.6. Human Readout “Real-World” Subsets

All images in the subset of the test dataset were presented in random order to two radiologists with extensive experience in the assessment of breast imaging examinations (J.W.: 6 years and A.B.: 16 years of experience). Both readers were blinded to patient information and rated each image of the BCT examination individually, according to the provided density atlas by Wieler et al. [18] The difference in image evaluation in our study compared to Wieler et al. was that only raw images in the coronal plane were used to train the dCNN and for the human read-out. The classification of the dataset of both readers served as a standard for the evaluation of the classification accuracy of the dCNN and was used for computation of inter-reader agreement between both readers.

### 2.7. Statistical Analyses

Statistical analysis was performed using the SPSS software package (SPSS version 23, International Business Machines Corp., Armon, NY, USA).

The metrics of the confusion matrices on the test dataset were quantified to assess the overall performances of the dCNN as compared to the assessment of the radiology resident, which served as the ground-truth.

For assessment of inter-reader agreement of the human readout, the intraclass correlation coefficient (ICC) between dCNN and both readers were calculated. According to Kundel and Polansky [23] and Landis and Koch [24], an ICC greater than 0.80 was considered to be indicative of “almost perfect agreement”. Inter-reader reliabilities of the dCNN and both readers were assessed by calculating kappa coefficients evaluated according to Cohen [25], whereas kappa values of 0.61–0.80 were considered substantial and kappa values of 0.81–0.90 were considered almost perfect.

The diagnostic performance of the dCNN compared to the human readout was assessed by conducting a receiver operating characteristics (ROC) analysis. For this purpose, the multiple classification of the test dataset into four categories (A, B, C and D) was translated into a binary classification system, splitting the BCT exams in two groups with high BCTD (category C and D) and lower BCTD (category A and B). Diagnostic accuracies were expressed as the area under the curve (AUC) and compared with DeLong’s nonparametric test [26] between the two readers and the dCNN. All tests were two tailed with *p*-values of <0.1.

## 3. Results

### 3.1. Patient Selection and Image Processing

#### 3.1.1. Patient Cohort

A total of 520 women received a BCT examination in our institution between January and August 2021. Based on the exclusion criteria, patient selection resulted in the inclusion of 317 healthy women corresponding to 634 BCT examinations. Mean age of the patient cohort was 55 years (34–83) with a median age of 55. The most common reason for BCT examination was screening mammography (*n* = 238), followed by mastodynia (*n* = 48). Follow-up examination of BI-RADS 3 or 4 findings (*n* = 11), the investigation of suspicious palpation findings (*n* = 9) or mastopathia (*n* = 4) were less common indications in this patient cohort. In our patient cohort, 214 (67.5%) women received an additional ultrasound, of which 103 were referred for breast cancer screening. In this patient cohort, 28 women (13%) received an additional ultrasound examination due to high density, although BD was assessed at level “B” according to our four-level density scale. This was because the strict application of the BD scale was not yet clinically established at this point. Breast density distribution was as follows: 58 (18.3%) women with density A, 118 (37.2%) women with density B, 83 (26.2%) women with density C and 58 (18.3%) with density D, categorized by human reading. Table 2 provides an overview of the patient cohort regarding BCTD and ultrasound examination. After image selection, pre-processing and data augmentation, a balanced dataset for training and validation, subdivided into the same four categories was available, containing 5109 pictures in total with an equal number from both left and right breast CT exams. Density distribution in the training and validation set was as follows: 1124 images category A (22%), 1840 images category B (36%), 1379 images category C (27%) and 766 images category D (15%).

#### 3.1.2. Accuracies in Training, Validation and “Real-World” Test Datasets

**Figure 2:**
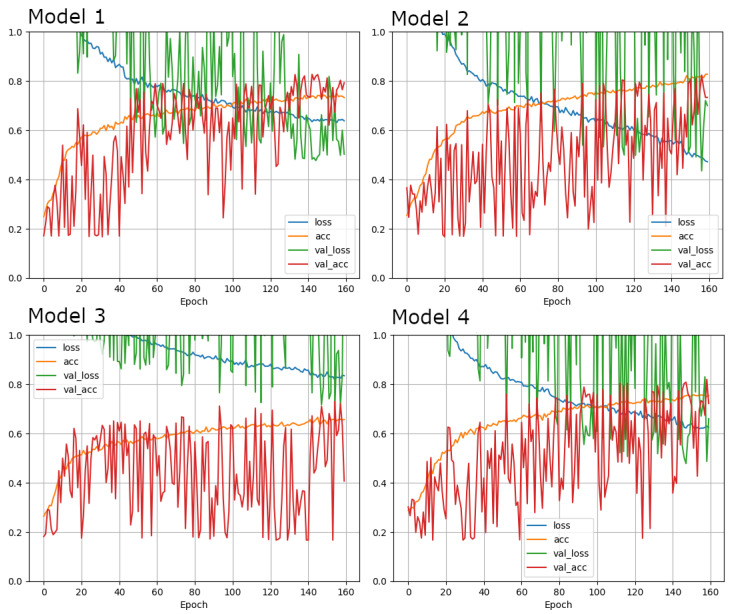
Training and validation accuracy and loss curves for the 4 different deep learning configurations. Model 1: Adam optimizer, Model 2: low resolution, Model 3: SGD optimizer and Model 4: cropped image. Highest accuracy was reached by Model 2.

The progression of the accuracies on training and validation for all four models are depicted in Figure 2. For models 2 and 3, accuracies were higher on the training sets compared to the validation datasets, whereas for models 1 and 4, accuracies were higher on the validation sets, which may be due to the relatively small batch size. Model 1 was the fastest learning model, reaching its maximum on the test set at epoch 150, with an accuracy of 74.1% and on the validation set at epoch 146, with an accuracy of 82.5%. Model 2 showed the highest overall accuracies, but also the slowest learning rate, reaching the highest accuracy on the training set at epoch 160 with 82.7% and on the validation set at epoch 156 with 82.3%. Overall performance on the training set of models 3 and 4 were comparable to each other (Model 3 at epoch 150: 74.1%; Model 4 at epoch 156: 75.5%), whereas Model 4 showed a substantially higher accuracy on the validation set (Model 3 at epoch 155: 72.9%; Model 4 at epoch 159: 81.9%).

The “real-world” test dataset of 480 images was applied to evaluate the four trained dCNN models on data that was not previously used for training. The distribution of density levels in the test dataset was the same as in the training and validation datasets: A: 106 images (26%), B: 173 images (36%), C: 108 images (23%); D: 73 images (15%). Again model 2, which differed from the other models by its lower input shape of 256 × 256 pixels compared to the 512 × 512-pixel matrices of the other models, exhibited the best overall performance with and accuracy of 85.8%. In model 1 and 4, the accuracy on the “real world” tests dataset was almost the same with 80.4% vs. 80.2%, showing that cropping of the center of image does not result in superior accuracy. Model 3 being the only model using SGD as an optimizer instead of Adam exhibited the lowest accuracy on the “real world” data with 73.5%. Table 3 shows the confusion matrices of the “real-world” test dataset for the four models compared to human reading as a ground-truth. 

#### 3.1.3. Human Readout

**Figure 3:**
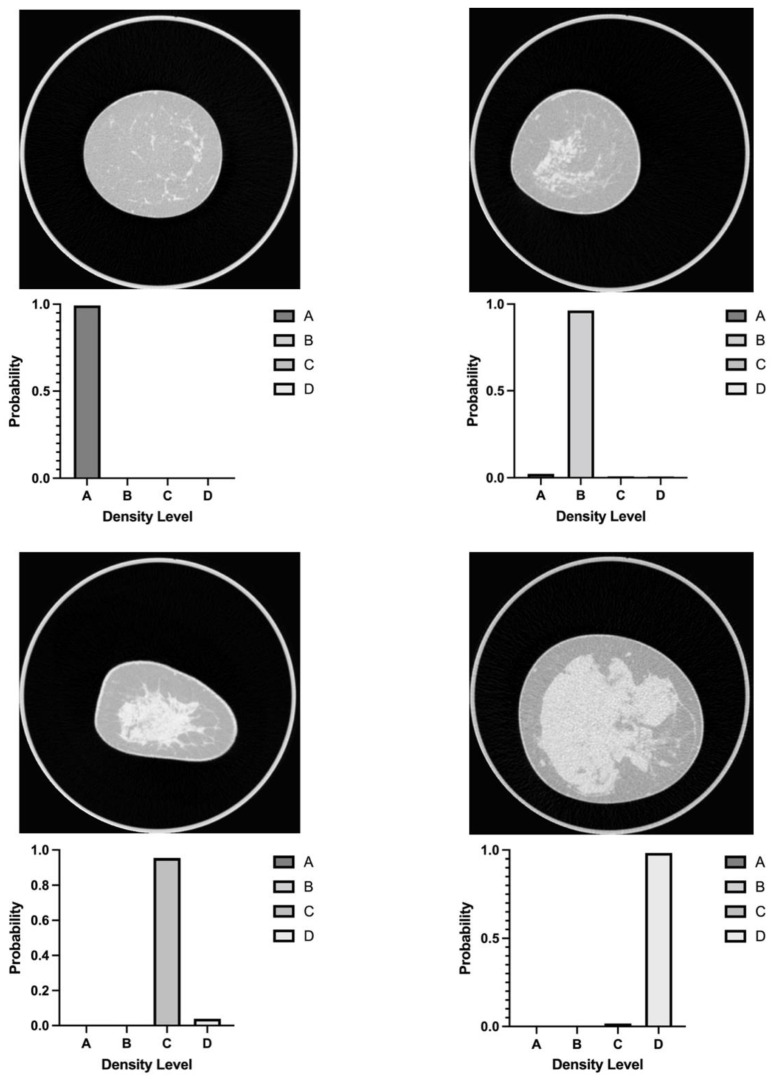
Example dCNN evaluations for breast-CT images showing different density levels, one example for each category.

**Figure 4:**
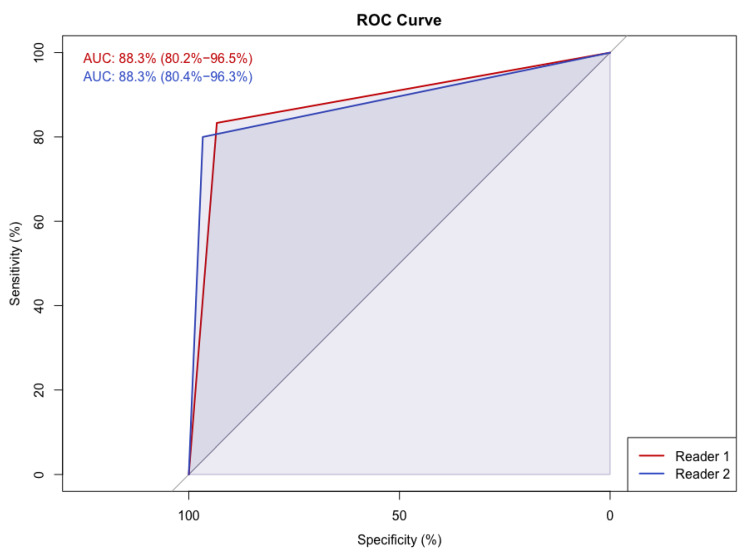
Receiver Operating Characteristics (ROC) Analysis for the Human Readout compared to the dCNN (Model 2) using a binary classification system: 0 = lower density (level A and B), 1 = high density (level C and D). Diagnostic Accuracy is expressed as the area under the curve (AUC).

Table 4 presents the classification results of the two experienced human readers in comparison to model 2, which exhibited the best overall performance among the dCNN models. Moreover, Figure 3 shows the classification probabilities for four example images of the test dataset from different categories calculated by the dCNN. Intraclass correlation coefficient (ICC) between dCNN and the two readers was 0.92 (95% CI 0.88, 0.95) reflecting an almost perfect interrater correlation. Kappa values between each pair of readers and dCNN, respectively, are shown in Table 5. Inter-reader reliability was substantial between both readers (0.73) as well as between human readers and dCNN (reader 1: 0.71; reader 2: 0.76) with all calculated *p*-values <0.001. In comparison, intra-reader reliability between the first human readout which served as the ground-truth and the two expert readers was almost perfect (reader 1: 0.87; reader 2: 0.82), as well as compared to the dCNN (0.84) (Table 5). Consensus between reader 1 and the ground-truth was better compared to the dCNN, whereas classifications of reader 2 showed better agreement with the dCNN. In all statistical comparisons, *p*-values were below significance level of 0.1 (*p* < 0.001 in each case). Inter-reader reliability between reader 1 and 2 for classification in either low density (A/B) not requiring additional ultrasound and high density (C/D) corresponding to the recommendation for additional ultrasound was almost perfect (kappa value 0.8). Diagnostic performance between the two readers and the dCNN showed very good correspondence with an AUC of 0.89 (95% CI 0.79, 0.98) for both readers, as depicted in Figure 4.

## 4. Discussion

Spiral breast-CT is a new and promising technique in breast cancer detection. The main advantages are its high isotropic spatial resolution, allowing three-dimensional imaging without tissue overlay and improved patient comfort due to the lack of breast compression. However, analogous to conventional mammography, breast-CT requires an additional ultrasound examination in patients with dense breasts to increase the cancer detection rate. In the present study, we were able to show that Artificial Intelligence in the form of a trained deep convolutional neural network (“deep learning”) can be used to classify breast density accurately and reliably in a BCT examination, allowing for standardized decision-making as to whether an additional ultrasound is required or not. By the proposed technique, the workflow in BCT examinations will be improved; thereby increasing the clinical applicability of this new breast imaging modality.

During and following conventional mammography, 35% percent of women experience discomfort and up to six percent of women report frank pain [27]. Therefore, up to 46% of women do not attend follow-up mammography due to negative and painful experience in their first mammography examination [28]. In our patient cohort 44 women of the included 317 patients corresponding to 13.8% of the patient cohort chose BCT instead of conventional mammography because of substantial pain in previous mammography screening examinations. To this date, BCT is the only modality to detect microcalcifications and soft tissue lesions without patient discomfort caused by breast compression. The breast-CT imager used in our patient cohort is the first clinically approved dedicated spiral-CT for breast imaging, equipped with a single photon-counting detector, which is capable of 3D imaging of the breast with a similar or even lower radiation dose than conventional mammography screening [16].

Although sensitivity is reported to be higher in BCT compared to conventional mammography, false-negative examinations due to tissue overlay are still an issue in breast cancer screening carried out with BCT [16]. The fact, that glandular tissue and breast lesions have the same or similar density, may impede lesion detection, especially in dense breast tissue. Therefore, a system for standardized reporting of BCTD as a surrogate for the sensitivity of the examination analogous to the ACR BI-RADS scale is greatly needed. Wieler et al. [18] proposed a four-level classification system (A to D) intended for human reading with the relevant features amount of breast parenchyma, distribution of breast parenchyma and visibility of fatty septae between glandular tissue, distinguishing the different classes. Earlier studies by Stomper et al. regarding the breast density distribution in the female population in conventional mammography reported similar distribution as in our patient cohort. In fact, the selection of categories A (partial or complete involution with every lesion visible) and D (very dense tissue with restricted visibility of even large lesions) is often straight forward. However, the majority of women are classified into categories B (scattered glandular tissue, no ultrasound needed) and C (heterogenous dense glandular tissue, additional ultrasound needed) [29]. The distinction between categories B and C is more difficult, requires more expertise of human readers in BCT evaluation and is prone to high observer variability [30,31]. The use of a dCNN for a standardized and observer independent classification of parenchymal density in BCT can be a supporting tool for a more reliable decision-making on supplementary ultrasound and can also improve the workflow. The performance of our dCNN compared to the human readout showed very good performance with substantial inter-reader agreement. Therefore, the implementation of the proposed dCNN into the clinical workflow may substantially reduce the inter-reader, as well as the intra-reader variability. Compared to conventional mammography with a typical volume of four single views, spiral breast CT can achieve a maximum volume of around 3500 images (including both, soft-tissue reconstructions and high-resolution images), resulting in a substantially longer evaluation time. Automated classification of BCTD by using a dCNN may result in reduced reading time of the radiologist, in addition to a standardized decision.

In our study, we propose an approach to determine breast density, according to Wieler et al. [18], using a deep convolutional neural network (dCNN), which is the most powerful machine learning algorithm for the classification of radiological images, however, it also requires a very large amount of data to reach satisfactory accuracy levels. Our four evaluated dCNN configurations were trained with approximately 6000 images of spiral breast-CT, which were labelled according to breast density by human reading. The best performing dCNN configuration (model 2) reached an average training accuracy of about 86%, using a lower input 256 × 256 matrix size compared to the other models, implying that high spatial resolution is not required for standardized decision-making. Therefore, the decision-making of our dCNN seems less linked to the fatty-septae criteria compared to human reading. This finding is somewhat surprising as in the human labeling, the fatty-septae criteria was regarded as a very important feature. A hypothesis on the reason why the dCNN is less dependent on the fatty-septae criteria may be that the fatty-septae are strongly correlated to other imaging features and therefore, may be substituted by the dCNN in the training. Unfortunately, one of the shortcomings of deep learning is that the classification decisions cannot be traced back to certain features, which is, e.g., possible in less sophisticated machine learning algorithms, such as decision trees or random forests.

The performance of our dCNN compared to the human readout showed very good performance with substantial inter-reader agreement. Therefore, the implementation of the proposed dCNN into the clinical workflow may not only reduce the inter-reader variability and intra-reader variability. Moreover, breast density is known to be linked to breast cancer risk, and an objective evaluation of BCTD by machine learning approach allows for a more accurate and reliable calculation of the breast cancer risk in the individual patient.

There are several limitations to this study. First, this was a single centre retrospective study, including only a small number of patients receiving BCT examination in our institution between January and August 2021. Second, because of the limited amount of data available in this relatively new imaging modality, multiple BCT images of the same patient were taken from different locations, potentially causing a bias of redundancy. Unlike Saffari et al. we did not use synthetic data generated by GANs, although our study focused on the feasibility of application of artificial intelligence for the assessment of BD in novel breast-CT devices [22]. Third, only representative pictures of breast density were taken, whereas images also containing silicone implants, pectoralis muscle or bones were omitted. Therefore, no information was obtained concerning whether BCTD can be assessed in images comprising additional structures. Fourth, only coronal images were used to train the machine learning algorithm. In “real world” also multiplanar reconstructions may be used by the radiologist for even more precise image assessment. Five, although we used a classification atlas based on lesion visibility, assessing lesion detection by the dCNN was out of the scope of this study. Sixth, only four different configurations of a dCNN were tested, and we cannot exclude the fact that other configurations may result in even higher accuracies.

In conclusion, we were able to train a deep Convolutional Neural Network which will allow the accurate, standardized and observer-independent classification of breast density in new photon-counting spiral breast CT, according to a four-level density atlas, analogous to the ACR BI-RADS classification system. The implementation of the dCNN into the clinical workflow may help improve the diagnostic accuracy and reliability of mammographic breast density assessment in the clinical routine by simultaneously reducing evaluation time.

## Figures and Tables

**Table 1:** Characteristics for each Model of the Deep Convolutional Neural Network.

	Model 1	Model 2	Model 3	Model 4
**Architecture**	brayZNet	brayZNet	brayZNet	brayZNet
**Learning rate**	1 × 10^−5^	1 × 10^−5^	1 × 10^−5^	1 × 10^−5^
**Loss function**	Cross entropy	Cross entropy	Cross entropy	Cross entropy
**Optimizer**	Adam	Adam	SGD	Adam
**Augmentation**	{‘zooming’: 0.1, ‘rotation’: 45.0, ‘horizontal_shift’: 0.1, ‘vertical_shift’: 0.1, ‘brightness’: 0.0}	{‘zooming’: 0.1, ‘rotation’: 45.0, ‘horizontal_shift’: 0.1, ‘vertical_shift’: 0.1, ‘brightness’: 0.0}	{‘zooming’: 0.1, ‘rotation’: 45.0, ‘horizontal_shift’: 0.1, ‘vertical_shift’: 0.1, ‘brightness’: 0.0}	{‘zooming’: 0.1, ‘rotation’: 45.0, ‘horizontal_shift’: 0.1, ‘vertical_shift’: 0.1, ‘brightness’: 0.0}
**Epochs**	160	160	160	160
**Batch size**	8	8	8	8
**Dropout**	0.5	0.5	0.5	0.5
**Input Shape**	[512, 512, 1]	[256, 256, 1]	[512, 512, 1]	[512, 512, 1]
**Cropping**	None	None	None	[0.12826739057573872, 0.8474049572056288, 0.200998651126856, 0.8363919170216573]
**Dense layers**	2	2	2	2
**Units in layer**	128	128	128	128
**Regularization**	l1 = 1 × 10^−6^, l2 = 1 × 10^−6^	l1 = 1 × 10^−6^, l2 = 1 × 10^−6^	l1 = 1 × 10^−6^, l2 = 1 × 10^−6^	l1 = 1 × 10^−6^, l2 = 1 × 10^−6^
**Test accuracy**	0.8041666746139526	0.8583333492279053	0.7354166507720947	0.8020833134651184

**Table 2:** Patient overview regarding breast density distribution and ultrasound examinations, with n describing the total number of patients.

	Density Level	Ultrasound (US)		Reason for US Examination	
		Yes	No	Density	Other
**A**	58	14	44	0	14
**B**	118	64	54	28	36
**C**	83	80	3	57	23
**D**	58	56	2	43	13
**Total n**	317	214	103	128	86

**Table 3:** Confusion matrices of the “real-world” dataset compared to the assessment of the radiologist as the ground-truth. Numbers in bold highlight the correctly assessed images.

	Predicted Density Level																
		Model 1				Model 2				Model 3				Model 4			
		A	B	C	D	A	B	C	D	A	B	C	D	A	B	C	D
**Density level (ground truth)**	**A**	**91**	15	0	0	**90**	15	1	0	**93**	13	0	0	**63**	43	0	0
	**B**	19	**131**	23	0	12	**142**	19	0	31	**129**	10	3	4	**154**	15	0
	**C**	0	25	**101**	2	0	11	**112**	5	1	38	**88**	1	0	20	**106**	2
	**D**	0	0	10	**63**	0	1	4	**68**	0	1	29	**43**	0	0	11	**62**

**Table 4:** Assessment of breast-CT density of two readers and dCNN Model 2 in 60 images.

	dCNN	Reader 1	Reader 2	
**A**	14	15	16	**Low density**
**B**	16	18	19	
**C**	15	17	10	**High density**
**D**	15	10	15	

**Table 5:** Kappa values between each pair of both readers, “ground-truth” and dCNN Model 2 using a four-level density scale. According to Landis and Koch: 0.6 to 0.8 substantial and >0.8 almost perfect agreement.

	Ground-Truth	dCNN	Reader 1	Reader 2
**Ground-Truth**		0.84	0.87	0.82
**dCNN**			0.71	0.73
**Reader 1**				0.73
**Reader 2**				

## Data Availability

The data presented in this study are available on request from the corresponding author. The data are not publicly available due to privacy restrictions.

## Supplementary Materials

The following are available online at https://www.mdpi.com/article/10.3390/diagnostics12010181/s1, Model of the dCNN architecture.

## References

[1] Moreno-Aspitia A., Advani P. (2014). Current strategies for the prevention of breast cancer. Breast Cancer Targets Ther..

[2] Plevritis S.K., Munoz D., Kurian A.W., Stout N.K., Alagoz O., Near A.M., Lee S.J., Broek J.J.V.D., Huang X., Schechter C.B. (2018). Association of Screening and Treatment with Breast Cancer Mortality by Molecular Subtype in US Women, 2000–2012. JAMA J. Am. Med Assoc..

[3] Boyd N.F., Martin L.J., Sun L., Guo H., Chiarelli A., Hislop G., Yaffe M., Minkin S. (2006). Body Size, Mammographic Density, and Breast Cancer Risk. Cancer Epidemiol. Biomark. Prev..

[4] Kamangar F., Dores G.M., Anderson W.F. (2006). Patterns of Cancer Incidence, Mortality, and Prevalence Across Five Continents: Defining Priorities to Reduce Cancer Disparities in Different Geographic Regions of the World. J. Clin. Oncol..

[5] Lam P.B., Vacek P.M., Geller B.M., Muss H.B. (2000). The association of increased weight, body mass index, and tissue density with the risk of breast carcinoma in Vermont. Cancer.

[6] Burton A., Maskarinec G., Perez-Gomez B., Vachon C., Miao H., Lajous M., López-Ridaura R., Rice M., Pereira A., Garmendia M.L. (2017). Mammographic density and ageing: A collaborative pooled analysis of cross-sectional data from 22 countries worldwide. PLoS Med..

[7] Spak D., Plaxco J., Santiago L., Dryden M., Dogan B. (2017). BI-RADS ® fifth edition: A summary of changes. Diagn. Interv. Imaging.

[8] Hollingsworth A.B. (2019). Redefining the sensitivity of screening mammography: A review. Am. J. Surg..

[9] Kolb T.M., Lichy J., Newhouse J.H. (2002). Comparison of the Performance of Screening Mammography, Physical Examination, and Breast US and Evaluation of Factors that Influence Them: An Analysis of 27,825 Patient Evaluations. Radiology.

[10] Wöckel A., Festl J., Stüber T., Brust K., Krockenberger M., Heuschmann P.U., Jírů-Hillmann S., Albert U.-S., Budach W., Follmann M. (2018). Interdisciplinary Screening, Diagnosis, Therapy and Follow-up of Breast Cancer. Guideline of the DGGG and the DKG (S3-Level, AWMF Registry Number 032/045OL, December 2017)—Part 2 with Recommendations for the Therapy of Primary, Recurrent and Advanced Breast Cancer. Geburtshilfe Frauenheilkd..

[11] Berger N., Marcon M., Frauenfelder T., Boss A. (2020). Dedicated Spiral Breast Computed Tomography with a Single Photon-Counting Detector. Investig. Radiol..

[12] Berger N., Marcon M., Saltybaeva N., Kalender W.A., Alkadhi H., Frauenfelder T., Boss A. (2019). Dedicated Breast Computed Tomography with a Photon-Counting Detector: Initial Results of Clinical In Vivo Imaging. Investig. Radiol..

[13] Wienbeck S., Fischer U., Luftner-Nagel S., Lotz J., Uhlig J. (2018). Contrast-enhanced cone-beam breast-CT (CBBCT): Clinical performance compared to mammography and MRI. Eur. Radiol..

[14] Li H., Yin L., He N., Han P., Zhu Y., Ma Y., Liu A., Lu H., Gao Z., Liu P. (2019). Comparison of comfort between cone beam breast computed tomography and digital mammography. Eur. J. Radiol..

[15] Shim S., Saltybaeva N., Berger N., Marcon M., Alkadhi H., Boss A. (2020). Lesion Detectability and Radiation Dose in Spiral Breast CT With Photon-Counting Detector Technology. Investig. Radiol..

[16] Wienbeck S., Uhlig J., Luftner-Nagel S., Zapf A., Surov A., von Fintel E., Stahnke V., Lotz J., Fischer U. (2017). The role of cone-beam breast-CT for breast cancer detection relative to breast density. Eur. Radiol..

[17] Ma Y., Cao Y., Liu A., Yin L., Han P., Li H., Zhang X., Ye Z. (2019). A Reliability Comparison of Cone-Beam Breast Computed Tomography and Mammography: Breast Density Assessment Referring to the Fifth Edition of the BI-RADS Atlas. Acad. Radiol..

[18] Wieler J., Berger N., Frauenfelder T., Marcon M., Boss A. (2021). Breast density in dedicated breast computed tomography. Medicine.

[19] Becker A., Marcon M., Ghafoor S., Wurnig M.C., Frauenfelder T., Boss A. (2017). Deep Learning in Mammography. Investig. Radiol..

[20] Ciritsis A., Rossi C., De Martini I., Eberhard M., Marcon M., Becker A.S., Berger N., Boss A. (2018). Determination of mammographic breast density using a deep convolutional neural network. Br. J. Radiol..

[21] Ciritsis A., Rossi C., Eberhard M., Marcon M., Becker A., Boss A. (2019). Automatic classification of ultrasound breast lesions using a deep convolutional neural network mimicking human decision-making. Eur. Radiol..

[22] Saffari N., Rashwan H., Abdel-Nasser M., Singh V.K., Arenas M., Mangina E., Herrera B., Puig D. (2020). Fully Automated Breast Density Segmentation and Classification Using Deep Learning. Diagnostics.

[23] Kundel H.L., Polansky M. (2003). Measurement of Observer Agreement. Radiology.

[24] Landis J.R., Koch G.G. (1977). The Measurement of Observer Agreement for Categorical Data. Biometrics.

[25] Cohen J. (1968). Weighted kappa: Nominal scale agreement provision for scaled disagreement or partial credit. Psychol. Bull..

[26] Delong E.R., Delong D.M., Clarke-Pearson D.L. (1988). Comparing the Areas under Two or More Correlated Receiver Operating Characteristic Curves: A Nonparametric Approach. Biometrics.

[27] Rutter D.R., Calnan M., Vaile M.S., Field S., Wade K.A. (1992). Discomfort and pain during mammography: Description, prediction, and prevention. BMJ.

[28] Whelehan P., Evans A., Wells M., MacGillivray S. (2013). The effect of mammography pain on repeat participation in breast cancer screening: A systematic review. Breast.

[29] Stomper P.C., D’Souza D.J., DiNitto P.A., Arredondo M.A. (1996). Analysis of parenchymal density on mammograms in 1353 women 25–79 years old. Am. J. Roentgenol..

[30] Ekpo E.U., Ujong U.P., Mello-Thoms C., McEntee M.F. (2016). Assessment of Interradiologist Agreement Regarding Mammographic Breast Density Classification Using the Fifth Edition of the BI-RADS Atlas. Am. J. Roentgenol..

[31] Winkel R.R., Von Euler-Chelpin M., Nielsen M., Diao P., Nielsen M.B., Uldall W.Y., Vejborg I. (2015). Inter-observer agreement according to three methods of evaluating mammographic density and parenchymal pattern in a case control study: Impact on relative risk of breast cancer. BMC Cancer.

