# Supplementary figures and images for: Applied Machine Learning in Spiral Breast-CT: Can We Train a Deep Convolutional Neural Network for Automatic, Standardized and Observer Independent Classification of Breast Density?
Source: Diagnostics (Basel). 2022 Jan 13;12(1):181. doi: 10.3390/diagnostics12010181 (PMC8775263; doi:10.3390/diagnostics12010181)

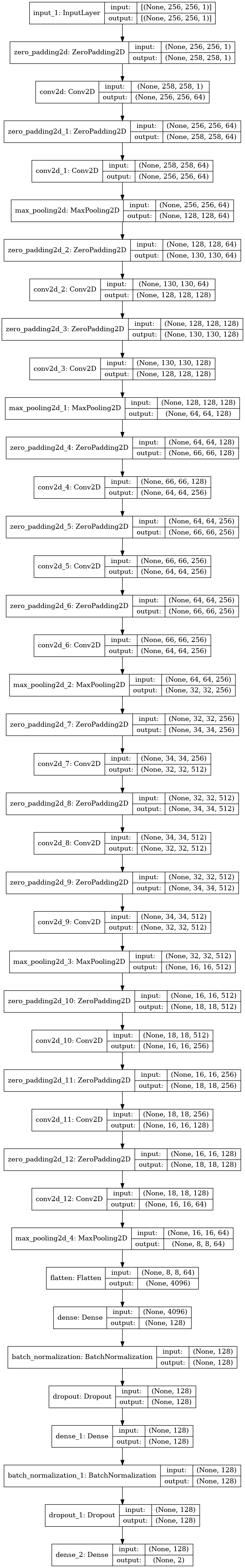

Supplement: Supplementary file 1 [file diagnostics-12-00181-s001.zip › diagnostics-1484109-supplementary.png]
